# Unveiling Apple Diversity: The Quality of Juice Produced from Old vs. Commercial Apple Cultivars

**DOI:** 10.3390/plants12213733

**Published:** 2023-10-31

**Authors:** Ante Lončarić, Ivana Flanjak, Tihomir Kovač, Ivana Tomac, Ana-Marija Gotal Skoko, Martina Skendrović Babojelić, Goran Fruk, Sanja Zec Zrinušić, Danijel Čiček, Jurislav Babić, Antun Jozinović

**Affiliations:** 1 Faculty of Food Technology Osijek, Josip Juraj Strossmayer University of Osijek, Franje Kuhača 18, 31000 Osijek, Croatia; ante.loncaric@ptfos.hr (A.L.); ivana.flanjak@ptfos.hr (I.F.); ivana.tomac@ptfos.hr (I.T.); amgotal@ptfos.hr (A.-M.G.S.); sanja.zec@ptfos.hr (S.Z.Z.); jurislav.babic@ptfos.hr (J.B.); antun.jozinovic@ptfos.hr (A.J.); 2 Faculty of Agriculture, University of Zagreb, Svetošimunska Cesta 25, 10000 Zagreb, Croatia; mskendrovic@agr.hr (M.S.B.); gfruk@agr.hr (G.F.); 3 Croatian Agency for Agriculture and Food, Center of Pomology and Vegetable Crops, Gorice 68b, 10000 Zagreb, Croatia; danijel.cicek@hapih.hr

**Keywords:** old apple cultivars, apple juice, polyphenol profile, antioxidant activity

## Abstract

This research is focused on comparing the compositions of juice produced from old and commercially grown apple cultivars. We examined factors such as pH, total acids, soluble dry matter, polyphenol profile, and antioxidant activity, which impact the attributes, safety, shelf life, and nutritional value of the juice. Our analysis revealed differences between these two groups of cultivars. For instance, pH values ranged from 3.04 (in ‘Bobovec’) to 3.69 (in ‘Fuji’). The proportions of acids varied from 0.07 g/100 mL (in ‘Fuji’) to 0.19 g/100 mL (in ‘Wagener’). Soluble dry matter content ranged from 14.10% (in ‘Fuji’) to 18.50% (in ‘Kraljevčica’). We also observed variations in sugar content and composition among cultivars; for example, sucrose levels varied from 16.11 g/L (‘Fuji’) to 39.36 g/L (‘Golden Delicious). Glucose levels ranged from 4.95 g/L (‘Jonagold’) to 19.18 g/L (‘Fuji’), while fructose levels spanned from 50.78 g/L (‘Austrougarka’) to 427.97 g/L (‘Ilzer Rosenapfel’). Furthermore, old apple cultivars exhibited higher concentrations of phenols and flavonoids compared to commercial ones; we also noted significant variations in flavonol levels among different cultivars. The ‘Wagener’ and ‘Božićnica’ apple varieties had levels of myricetin measuring 0.53 and 0.52 µg/mL, respectively. On the other hand, ‘Bobovec’ stood out for its content of procyanidin B2 with a concentration of 422.61 µg/mL. When examining non-flavonoid compounds, it was found that old apple cultivars had higher concentrations of gallic acid, *trans*-ferulic acid, and chlorogenic acid. However, commercial cultivars showed dominance in caffeic and *p*-coumaric. Comparisons of antioxidant capacity using DPPH and ABTS assays clearly demonstrated the superiority of old apple cultivars. Overall, this study highlights the importance of utilizing apple cultivars for juice production. Their distinct compositions and higher antioxidant capacities contribute to potential health benefits. Preserving these cultivars for enhanced juice quality and nutritional value is encouraged. Further research could explore cultivation practices’ impact on composition and health benefits.

## 1. Introduction

Apple juice is one of the most popular beverages worldwide. It is valued for its taste, nutritional content, and potential health benefits [1]. The quality of apple juice depends on several factors, including the cultivar, agricultural practices, and processing techniques [2].

In recent years, there has been growing interest in comparing the quality of apples and apple products produced from old apple cultivars with those from commercial cultivars in terms of their bioactive composition, taste, and potential health benefits. Old apple cultivars, also known as heirloom or heritage cultivars, are cultivated for generations and are often locally adapted to specific regions [3]. Old apple cultivars garnered attention for their unique flavors, diverse genetic backgrounds, and potential health-promoting properties, such as their high levels of polyphenols and antioxidants. Furthermore, old apple cultivars, which are often better adapted to local conditions, can support a diverse range of flora and fauna, including beneficial insects and pollinators. This, in turn, can lead to the preservation of vital ecosystem services such as pollination, pest control, and nutrient cycling, all of which are essential for the long-term sustainability of agricultural systems [4]. This can help to support rural communities, preserve old agricultural practices, and encourage the continuation of local knowledge and cultural heritage associated with these old apple cultivars. In contrast, commercial apple cultivars are typically bred for their appearance, shelf life, and yield, sometimes at the expense of their nutritional value and taste [5,6]. The majority of phytochemicals found in apples are polyphenols, which belong to a subgroup of phytochemicals. Apples contain five primary groups of polyphenols, including flavonols (such as quercetin glycosides), flavanols (such as catechins, epicatechin, and procyanidins), phenolic acids (such as chlorogenic, gallic, and coumaric acids), dihydrochalcones (including phloretin glycosides), and anthocyanins (cyanidin) [5]. Polyphenols are bioactive compounds that possess antioxidant, anti-inflammatory, and antimicrobial properties [7]. Several studies have reported that old apple cultivars have higher concentrations of bioactive compounds compared to commercial cultivars. For instance, in our study conducted on the peels of old and new apple cultivars, we found that old Croatian apple cultivars had a higher level of polyphenolic compounds compared with new ones [8]. The most abundant polyphenols in old apple peel were chlorogenic acid, procyanidin B2, and epicatechin. Furthermore, the peel of the cultivars ‘Apistar’, ‘Bobovac’, and ‘Božićnica’ was highlighted as an important source of polyphenols. The study conducted by Butkeviciute et al. [9] highlights the variability of the qualitative and quantitative composition of phenolic compounds in Lithuanian heirloom apple cultivars. Phenolic compounds, which are responsible for potent biological effects such as antioxidant activity, were identified and quantified. The phenolics identified included flavan-3-ols (such as procyanidin B1, procyanidin B2, procyanidin C2, (+)-catechin and (−)-epicatechin), flavonols (such as rutin), and other compounds such as chlorogenic acids and phloridzin. In particular, flavan-3-ols accounted for 30% of the total quantity of phenolic compounds. Among the flavan-3-ols, (−)-epicatechin was the most dominant compound. The total quantity of the identified phenolic compounds ranged from 0.15 ± 0.01 mg/g to 3.82 ± 0.53 mg/g among different heirloom apple cultivars. The ‘Koštelė’ apple cultivar had the highest sum of the identified phenolic compounds (3.82 ± 0.53 mg/g). They concluded that the polyphenol profile in the Lithuanian apple cultivars was found to be higher than those in heirloom apple cultivars from Italy or Brazil but lower than those found in the Italian region of Piedmont. Therefore, the specific polyphenol profile of these heirloom apple cultivars may contribute to their unique nutritional and health-promoting qualities [9]. Furthermore, Kschonsek et al. [5] concluded in their study that the peel and flesh of old apple cultivars had a higher content of polyphenolic compounds and vitamin C, resulting in a higher antioxidant content (AOC) compared to new apple cultivars, therefore advising the consumption of apple with peel, due to its higher polyphenolic content and stronger AOC. Similarly, Veberic et al. [10] reported that old Slovenian apple cultivars contained significantly higher amounts of phenolic compounds compared to commercial cultivars, with chlorogenic acid, epicatechin, and procyanidin B2 being the most abundant. However, they emphasize that organically grown plants are exposed to different kinds of stress, which could lead to a response in which higher levels of polyphenols are synthesized. Additionally, the antioxidant capacity of apple juice has gained considerable attention due to its potential health benefits. Antioxidants in apple juice, primarily derived from polyphenols and flavonoids, have been associated with reduced risks of chronic diseases, such as cardiovascular disease, diabetes, and certain cancers [1,11,12]. Oszmiański et al. [13] demonstrated that the antioxidant potential of old Polish apple cultivars varied significantly, with cvs. ‘Roter Trier Weinapfel’, ‘Wintergoldparmane’, and ‘Horneburger Pfannkuchenapfel’ having the highest polyphenolic content. The sugar and organic acid composition of apple juice also plays a crucial role in its sensory characteristics and consumer preferences. Akagic et al. [14] observed that old apple cultivars had higher levels of total phenolics, organic acids, and sugars than their commercial counterparts. These differences in sugar and organic acid content can influence the taste, aroma, and overall quality of apple juice, potentially making old apple cultivars more attractive to consumers seeking unique and diverse flavors [15]. Moreover, the diversity of old apple cultivars may contribute to the production of a variety of apple juices, each with its own distinctive flavor and nutritional value. In addition to the unique taste profiles and potential health benefits associated with old apple cultivars, the utilization of these cultivars in juice production can have positive implications for sustainability and biodiversity.

The aim of this study is firstly: to comprehensively compare the quality of apple juices produced from old and commercial apple cultivars; secondly: to prove the hypothesis that old apple cultivars are superior in terms of bioactive components compared to commercial ones; and finally: to assess the potential of old apple cultivars for diversified juice production and conservation efforts.

## 2. Results and Discussion

The pH, total acids, and soluble dry matter are important parameters for apple juice as they affect its sensory quality, safety, and shelf life. pH determines the acidity level of the juice, which affects its taste, stability, and microbial growth. Total acids provide information about the acid composition and balance in the juice, which is crucial for its flavor and preservation. Soluble dry matter measures the amount of solids in the juice, which affects its sweetness, viscosity, and nutritional value. The results for the physicochemical composition of juices produced from old and commercial apple cultivars are presented in Table 1.

The comparison of juice produced from old and commercial apple cultivars revealed significant differences in pH value, total acids, and soluble dry matter. Our results showed that the pH value ranged from 3.04 (‘Bobovec’) to 3.69 (‘Fuji’). This finding is in agreement with similar studies conducted on apple juice, where the pH values were reported to range from 3.5 to 4.0 [16,17]. The proportion of total acids varied from 0.07 g/100 mL expressed as malic acid (‘Fuji’) to 0.19 g/100 mL expressed as malic acid (‘Wagener’). Our findings are consistent with previous studies that reported the total acid content of apple juice ranged from 0.06 to 0.18 g/100 mL [17,18]. Regarding the proportion of soluble dry matter expressed in °Brix, our results showed that it ranged from 14.1 (‘Fuji’) to 18.5 (‘Kraljevčica’). This finding is in agreement with previous studies conducted on apple juice, which reported the soluble dry matter content to range from 12.6 to 15.5 °Brix [18].

The results of sugar content and composition presented in Table 2 showed significant differences in the proportions of these sugars across the different cultivars. The proportion of sucrose in the juice samples ranged from 16.11 ± 1.67 g/L (‘Fuji’) to 39.36 ± 0.65 g/L (‘Golden Delicious’). The proportion of glucose ranged from 4.95 ± 0.30 g/L (‘Jonagold’) to 19.18 ± 0.00 g/L (‘Fuji’), while the proportion of fructose ranged from 50.78 ± 0.22 g/L (‘Austrougarka’) to 27.97 ± 0.34 g/L (‘Ilzer Rosenapfel’). The differences in sugar content may be attributed to various factors, including genetic and environmental factors and differences in cultivation practices [19]. As apples ripen, their starch content is converted into simple sugars, primarily glucose and fructose, through the action of enzymes such as amylase and invertase. Sucrose, which is present in apples in small quantities, is also hydrolysed into glucose and fructose during ripening [20,21]. Our findings are consistent with previous studies that reported a lower amount of sucrose content and higher amounts of fructose content in investigated apple cultivars [21,22]. In terms of the desirable sugars in apple juice, fructose is preferred over sucrose, as it has a sweeter taste and is more readily assimilated by the human body. Additionally, the ratio of glucose to fructose is an important determinant of the taste and sweetness of apple juice, with a higher fructose-to-glucose ratio resulting in a sweeter taste. Our findings suggest that, taking into account the fructose-to-glucose ratio of 7.48, followed by ‘Golden Delicious’ 5.93 and ‘Austrougarka’ 5.11 have the highest fructose-to-glucose ratio, resulting in a sweeter taste compared with other investigated apple juice. Furthermore, the cultivars with the highest fructose-to-glucose ratio, such as ‘Golden Delicious’ and ‘Austrougarka’, are likely to have a sweeter taste compared to other apple varieties. This is due to the higher concentration of fructose, which is a sweet-tasting sugar, compared to glucose. The ratio of fructose to glucose in ‘Golden Delicious’ was found to be 5.93, making it an excellent choice for those who prefer a sweeter apple juice. Interestingly, ‘Fuji’, despite having a high fructose content, has a relatively lower fructose-to-glucose ratio of 1.92.

The total phenol and flavonoid contents of juices produced from old and commercial apple cultivars are shown in Table 3. The obtained results showed that juices from old apple cultivars had a higher content of total polyphenols and flavonoids compared to those produced from commercial cultivars. The observed range of total polyphenols in juices produced from old apple cultivars (115.09 ± 0.98 to 501.62 ± 0.74 mg/L) and total flavonoids (29.21 ± 1.28 to 165.58 ± 2.21 mg/L) was consistent with previous studies [18,23,24,25,26]. The content of total polyphenols and flavonoids in juices is highly influenced by the apple cultivar used for their production. The obtained results are consistent with the findings reported in previous studies [26,27]. The higher content of polyphenols and flavonoids in juices produced from old apple cultivars can be attributed to the fact that old cultivars are grown in a more natural environment and subjected to fewer treatments with synthetic chemicals, such as pesticides and herbicides. In contrast, commercial cultivars are often grown using intensive agricultural practices that may result in a reduction in the content of these compounds [8,28]. The correlation between total polyphenols and flavonoids in juices has been investigated in several studies. A positive correlation between total polyphenols and flavonoids was reported by several authors [29]. These findings are in agreement with the results obtained in our study. The observed correlation between total polyphenols and flavonoids can be explained by the fact that flavonoids are a subclass of polyphenols, and thus their content is strongly related.

The statistical analysis of the data revealed significant differences (*p* ≤ 0.05) in the content of flavonols and their glycosides among different apple cultivars (Table 4). However, the studied flavonols were in the range of concentration reported by Kahle et al. [30]. The highest levels of myricetin were observed in ‘Wagener’, ‘Božićnica’, and ‘Kanadska Reneta’, while the lowest were detected in ‘Kraljevčica’, ‘Ilzer Rosenapfel’, ‘Fuji’, ‘Idared’, and ‘Golden Delicious’. For quercetin-3-rutinoside, the highest content was measured in ‘Božićnica’, ‘Bobovec’, and ‘Wagener’, while the lowest was in ‘Čelenka’, ‘Ilzer Rosenapfel’, ‘Idared’, ‘Golden Delicious’, ‘Fuji’, and ‘Jonagold’. Quercetin-3-glucoside was the most abundant flavonoid in ‘Austrougarka’, ‘Bobovec’, ‘Zelenika’, and ‘Granny Smith’, while it was detected in low amounts in ‘Čelenka’, ‘Golden Delicious’, and ‘Idared’. The amounts of quercetin in tested juices were low; this is due to several factors that affect the extraction and stability of quercetin during juice processing. Lee et al. [31] found that the concentration of quercetin in apple juice decreased significantly after thermal treatment, such as pasteurization, due to its thermal degradation. The same was reported by Chen et al. [32]. The lowest levels or bellow limit of detection (<LOD) of quercetin were observed in ‘Fuji’, ‘Idared’, ‘Golden Delicious’, and ‘Granny Smith’, while the highest content was detected in ‘Austrougarka’, ‘Zelenika’, ‘Kanadska Reneta’, and ‘Božićnica’. The obtained results are in line with previous studies that also reported significant differences in the content of flavonols among different apple cultivars [28,33]. Flavonols are known for their beneficial effects on human health, including antioxidant, anti-inflammatory, and anticancer activities [34]. Therefore, the higher content of flavonols in some old apple cultivars, such as ‘Božićnica’, and ‘Bobovec’, may contribute to their higher nutritional value and health benefits compared to some commercial cultivars.

The content of procyanidins in juices produced from old and commercial apple cultivars is shown in Table 5. For procyanidin A2 content, the cultivar with the highest value was ‘Kanadska Reneta’ (2.04 ± 0.34 µg/mL), while the lowest value was found in ‘Čelenka’ (0.13 ± 0 µg/mL). Similarly, for procyanidin B1 content, ‘Ilzer Rosenapfel’ had the highest content (135.32 ± 1.4 µg/mL), while ‘Čelenka’ had the lowest content (8.55 ± 0.37 µg/mL). For procyanidin B2 content, ‘Bobovec’ had the highest content (422.61 ± 2.01 µg/mL), while ‘Čelenka’ had the lowest content (3.21 ± 0.15 µg/mL). The results obtained in this study are in line with previous research on the content of procyanidins in apple juices. For example, a study by Schempp et al. [35] found that the content of procyanidin B2 in apple juices ranged from 0.01 to 285.5 mg/L, depending on the apple cultivar. A study by Février et al. [36] reported that the content of total procyanidins in apple juice varied between 41 mg/L and 1013 mg/L. However, it is important to note that these studies used different analytical methods and apple cultivars, which can significantly affect the measured content of procyanidins. Procyanidins have been shown to have a range of health benefits, such as antioxidant and anti-inflammatory effects, and may also have potential for cancer prevention [37]. The results of this study suggest that old apple cultivars may be a better source of procyanidins compared to commercial cultivars. This is in line with previous research that has shown that old apple cultivars generally have higher levels of bioactive compounds compared to commercial cultivars [8,28,37,38].

The results of the measurement of flavanols and dihydrochalcones in juices produced from old and commercial apple cultivars are shown in Table 6. The highest catechin content was observed in the old cultivar ‘Bobovec’ (26.86 ± 1.08 µg/mL), which was significantly higher than in commercial cultivars such as ‘Golden Delicious’ (0.48 ± 0.11 µg/mL) and ‘Idared’ (1.04 ± 0.04 µg/mL). Similarly, ‘Bobovec’ showed the highest epicatechin content (57.18 ± 1.28 µg/mL) compared to other cultivars (*p* ≤ 0.05). This is in agreement with previous research that has demonstrated higher polyphenolic content in old apple cultivars compared to commercial ones [8,32,39]. Regarding epigallocatechin content, ‘Granny Smith’, a commercial apple cultivar, had the highest concentration (41.06 ± 7.92 µg/mL), while some old cultivars such as ‘Čelenka’ exhibited concentrations below the limit of detection (≤LOD). Phloridzin content was highest in ‘Wagener’ (11.4 ± 0.07 µg/mL), an old apple cultivar, and was significantly different from commercial cultivars such as ‘Golden Delicious’ (1.04 ± 0.01 µg/mL) (*p* ≤ 0.05). Previous studies have shown that phloridzin, an apple-specific dihydrochalcone, possesses strong antioxidant and anti-inflammatory properties [34]. Eisele et al. [19] studied the partial compositional characteristics of apple juice from 175 apple cultivars, and they reported that the content of phloridzin in apple juice varied between 0.9 mg/L and 120.3 mg/L. Furthermore, Schempp et al. [35] studied 16 juices produced from different apple cultivars and found that total flavanols ranged from 0.3 to 955.8 mg/L. The differences in polyphenolic content among apple cultivars can be attributed to genetic and environmental factors, as well as differences in cultivation practices [33].

The results of the measurement of non-flavonoids in juices produced from old and commercial apple cultivars are shown in Table 7. The results showed significant differences in the concentrations of gallic acid, *trans*-ferulic acid, chlorogenic acid, caffeic acid, and *p*-coumaric acid among the apple cultivars (*p* ≤ 0.05). The old apple cultivars in most cases exhibited higher concentrations of gallic acid, *trans*-ferulic acid, and chlorogenic acid compared to the commercial apple cultivars. The old apple cultivars (‘Ilzer Rosenapfel’, ‘Wagener’, and ‘Kraljevčica’) showed higher concentrations of gallic acid, ranging from 2.37 to 4.9 µg/mL, compared with commercial apple cultivars (‘Golden Delicious’ and ‘Granny Smith’), which had comparatively lower concentrations of gallic acid, ranging from 0.1 to 0.85 µg/mL. Chlorogenic acid, an essential phenolic compound contributing to the antioxidant properties of apple juice, was found in higher concentrations in old apple cultivars such as ‘Winter Banane’ (353.74 µg/mL) and ‘Bobovec’ (563.96 µg/mL). This observation aligns with earlier research that reported a higher chlorogenic acid content in old apple cultivars compared to commercial ones [40]. Trans-ferulic acid, an important phenolic acid for its potential health benefits, was detected at higher levels in some old apple cultivars, such as ‘Winter Banane’ (0.24 µg/mL) and ‘Kanadska reneta’ (0.42 µg/mL), as opposed to commercial cultivars such as ‘Golden Delicious’ (0.03 µg/mL) and ‘Granny Smith’ (0.02 µg/mL). In contrast, commercial cultivars exhibited higher concentrations of caffeic acid and *p*-coumaric acid. For instance, ‘Granny Smith’ had a caffeic acid concentration of 0.47 µg/mL and a *p*-coumaric acid concentration of 0.06 µg/mL, higher than most old apple cultivars. The results of this study highlight the importance of preserving and promoting the use of old apple cultivars in juice production due to their higher phenolic compound content and potential health benefits. Moreover, the findings contribute to the existing knowledge on the differences in phenolic content between old and commercial apple cultivars [3].

In the investigation conducted by Wojdyło et al. [41], a significant variation in the levels of polyphenolic compounds among different apple varieties was observed. Their findings underscored the pivotal role of genotype in shaping the profile of individual phenolic constituents within apples. The outcome of their study indicated that, predominantly, procyanidins, flavan-3-ols, and chlorogenic acid constituted the major fraction of polyphenolic constituents in apples, while anthocyanins and phloridzin were found to be present in lesser proportions. Significantly, the concentration of procyanidins/flavan-3-ols was identified as the highest contributor to in vitro antioxidant activity.

In the context of our study, a consensus can be drawn regarding the substantial representation of procyanidins within the overall polyphenolic content. Furthermore, both investigations underscore the discernible variability in the polyphenolic composition of apple cultivars, a variability contingent upon genetic and environmental influences. Our findings align with this premise, suggesting that older apple cultivars may exhibit heightened concentrations of specific polyphenolic compounds, including procyanidins, gallic acid, trans-ferulic acid, and chlorogenic acid. However, it is worth noting that this proclivity can be subject to variation contingent upon the specific apple varieties under consideration, as is evinced by the Wojdyło et al. [41] study, which occasionally reported analogous or even elevated levels of these bioactive compounds in newer apple varieties in comparison to their older counterparts.

Notably, both studies underscore the significance of polyphenolic compounds sourced from apples, particularly in the context of their potential health benefits, which encompass antioxidant and anti-inflammatory effects. Chlorogenic acid emerges as a preeminent polyphenolic constituent in both investigations, thereby underscoring its substantive role within the polyphenolic arsenal of apples. These findings contribute to a richer understanding of the multifaceted role of polyphenolic compounds in apples and their implications for human health.

The old apple cultivars, such as ‘Austrougarka’, ‘Bobovec’, and ‘Božićnica’, exhibited higher antioxidant capacities in both DPPH and ABTS assays compared to most commercial apple cultivars such as ‘Golden Delicious’, ‘Idared’, and ‘Jonagold’ (Table 8). These findings are consistent with previous research indicating that old apple cultivars generally possess higher levels of antioxidants than commercial cultivars [28,37,38,42]. Comparing the DPPH and ABTS assays, the values obtained from the ABTS assay were generally higher than those from the DPPH assay, which is in line with other studies that have found the ABTS assay to be more sensitive and thus yielding higher antioxidant capacity values. Nonetheless, both assays displayed a similar trend of higher antioxidant capacities in old apple cultivars compared to commercial ones. The observed differences in antioxidant capacities could have significant implications for the health benefits and nutritional value of apple juice. The higher antioxidant capacity in old apple cultivars may offer better protection against oxidative stress-related diseases. Furthermore, these results support the need for conserving and promoting old apple cultivars, as they could contribute to the development of high-quality apple products with potential health benefits [12].

## 3. Materials and Methods

### 3.1. Materials

**Figure 1:**
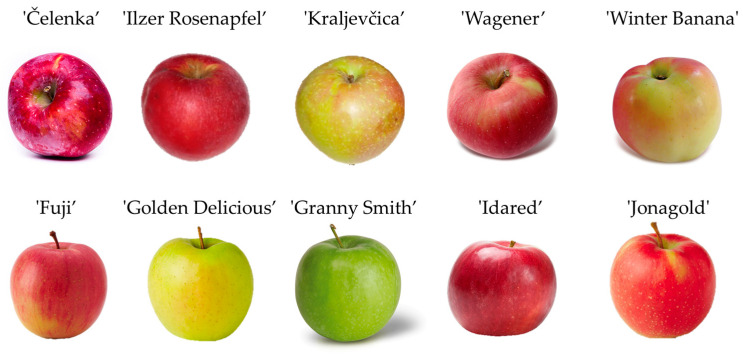
Apple cultivars used in experiment.

The old apple cultivars ‘Čelenka’, ‘Ilzer Rosenapfel’, ‘Kraljevčica’, ‘Wagener’, ‘Winter Banana’ were collected at Šašinovec, Croatia (45°85′00.3″ N, 16°17′75.2″ E); ‘Bobovec’, ‘Božićnica’, ‘Austrougarka’, ‘Kanadska Reneta’, ‘Zelenika’ at Perenci, Požega (45°23′45.0″ N 17°34′06.3″ E); and commercial apple cultivars, ‘Fuji’, ‘Golden Delicious’, ‘Granny Smith’, ‘Idared’, ‘Jonagold’ at Novaki Bistranski, Donja Bistra, Croatia (45°54′57.0″ N 15°52′56.0″ E). All studied apple cultivars (Figure 1) were authenticated by a pomologist and confirmed by 12 SSR markers.

### 3.2. Chemicals

For determination of individual polyphenols, standard stock solutions (2000–8000 µg/mL) were prepared in methanol using pure polyphenols, including myricetin, quercetin-3-rutinoside, quercetin-3-d-glucoside, quercetin, procyanidin A2, procyanidin B1, procyanidin B2, catechin, epicatechin, epigallocatechin, phloridzin, gallic acid, trans-ferulic acid, chlorogenic acid, caffeic acid, and p-coumaric acid, all of which were provided by Sigma-Aldrich (Chemie Check GmbH, Steinheim, Germany). From these prepared solutions, standard calibration curve solutions were then generated. The solutions were stored at −20 °C and protected from light. For the determination of sugars, a standard calibration curve was generated using sucrose, glucose, and fructose standards provided by Sigma-Aldrich (Chemie Check GmbH, Steinheim, Germany).

### 3.3. Juice Production

The cloudy apple juice was produced by crushing and pressing five kilograms of apples in the laboratory juicer. After the pressing, the juice was pasteurized at 80 °C for two minutes and filled in the appropriate packaging. The juice was produced from apples immediately after picking and stored at 4 °C prior to analysis.

### 3.4. Determination of Physico-Chemical Composition

The soluble dry matter of juices was measured using a table Abbe refractometer and expressed in Brix (°Brix). The pH value was measured with a pH meter from Mettler Tolledo Columbus, Ohio, SAD. The acids were measured by titration with 0.1 M NaOH and phenolphthalein as an indicator and expressed in g/100 mL as malic acid.

### 3.5. Determination of Sugars

The separation of carbohydrates (fructose, glucose, and sucrose) was performed on a Zorbax NH_2_ HPLC column (Agilent, USA, 4.6 *×* 250 mm, particle size 5 µm). Mobile phase (acetonitrile/water, 70/30) flow was 1 mL/min. The injection volume was 10 µL, and the column and refractive index detector temperatures were set at 45 °C. Carbohydrates were identified by their retention time, and the quantification was carried out through the external calibration method. The content of fructose, glucose, and sucrose in apple juices was expressed as grams of individual carbohydrate per liter of apple juice (g/L).

### 3.6. Determination of Polyphenols

The total polyphenol content was measured using the Folin–Ciocalteu method, with some modifications, as previously described in our work [43]. The absorbance was read at 765 nm using a spectrophotometer (Perkin Elmer, LAMBDA™ 365, Waltham, MA, USA). Each sample was measured in triplicate, and the average value was interpolated on a gallic acid calibration curve and expressed as mg/L.

The total flavonoid content was determined according to Makris et al. [39]. In summary, 0.5 mL of the extract was combined with 4 mL of distilled water. Then, 0.3 mL of a 5% NaNO_2_ solution was introduced and allowed to react for 5 min. Subsequently, 0.3 mL of a 10% AlCl_3_ solution was added, and the mixture was allowed to react for an additional 5 min. Finally, 2 mL of a 1 M Na_2_CO_3_ solution and 2.4 mL of distilled water were introduced into the reaction mixture. The absorbance at 510 nm was then measured against a blank. Each sample was analyzed in triplicate, and the results were calculated by interpolating them on a calibration curve using catechin as a reference standard. The results were expressed as milligrams of catechin equivalents per liter of apple juice (mg CE/L).

Individual polyphenols were quantified using high-performance liquid chromatography (HPLC) employing the Shimadzu HPLC instrument. The mobile phase was composed of two components: A (water with 1% formic acid) and B (methanol with 1% formic acid). The UV–vis absorption spectra for both the standards and the test samples were captured within the wavelength range of 190 to 600 nm. The identification of polyphenols encompassed five flavanols, four flavonols, five phenolic acids, two dihydrochalcones, and three anthocyanins, achieved through a comparison of their retention times and UV–vis spectra with those of pure standards. Detection was performed at wavelengths of 280, 320, 360, and 520 nm (Figure S1). The concentration of polyphenols was expressed in units of micrograms per milliliter (μg/mL).

The ABTS assay was conducted following the method of Arnao et al. [44], with slight modifications. A total of 3.2 mL of ABTS solution was mixed with 0.2 mL of apple extract, and the mixture was left for an hour and 35 min before measuring the absorbance at 734 nm. The results were expressed as mmol Trolox equivalents/L of sample. If the measured ABTS value was over the linear range of the standard curve, an additional dilution was required.

The DPPH• scavenging activity assay followed the method described by Brand-Williams et al. [41] with small modifications. Specifically, a mixture was prepared by combining 0.2 mL of apple extract with 2 mL of methanol and 1 mL of a DPPH ethanol solution (0.5 mM). Following a 15-min incubation period, the absorbance was recorded at 517 nm. The outcomes were quantified as mmol Trolox equivalents per liter of the sample. If the measured DPPH value exceeded the linear range of the standard curve, additional dilution was necessary. These techniques were utilized in the research to precisely assess the antioxidant activity of ABTS and DPPH• in apple extracts.

### 3.7. Statistical Analysis

Data presented in this work are expressed as the mean value ± SD (standard error of measurement) from three separate experiments. The experimental data were subjected to a one-way analysis of variance (ANOVA) and Fisher’s LSD was calculated to detect a significant difference between the mean values. Statistical analyses were performed using the Statistica 13.5 (TIBCO Software Inc., Palo Alto, CA, USA), and differences were considered statistically significant when the *p* value was <0.05.

## 4. Conclusions

This study provides a comprehensive comparison of juice produced from old and commercial apple cultivars, highlighting differences in their pH value, total acids, soluble dry matter, sugar content, and phenolic compound content. Old apple cultivars generally exhibited a higher content of total polyphenols, flavonoids, and procyanidins compared to commercial cultivars. The higher antioxidant capacities of old apple cultivars further contribute to their nutritional value and potential health benefits.

Our findings underscore the importance of preserving and promoting the use of old apple cultivars in juice production due to their potential superior health benefits. Additionally, these results contribute to the existing knowledge on the differences in chemical composition and bioactive compound content between old and commercial apple cultivars. This information can be valuable for apple growers, juice producers, and consumers who seek to make informed choices regarding apple cultivars and juice products with optimal nutritional and health-promoting properties.

Future research should explore the impact of various cultivation practices, environmental factors, and genetic traits on the chemical composition and bioactive compound content of apple cultivars. Moreover, further studies could investigate the potential health benefits associated with the consumption of apple juice produced from old cultivars, particularly concerning their antioxidant, anti-inflammatory, and anticancer activities. This information could provide valuable insights for the development of high-quality, health-promoting apple juice products and inform sustainable agricultural practices for apple cultivation.

## Figures and Tables

**Table 1:** Physicochemical composition of juices produced from old and commercial apple cultivars.

	pH	TA (g/100 mL as Malic Acid)	SDM (°Brix)
‘Wagener’	3.06 ± 0.00	0.19 ± 0.03	16.6 ± 0.01
‘Ilzer Rosenapfel’	3.26 ± 0.02	0.11 ± 0.02	17.5 ± 0.09
‘Winter Banane’	3.31 ± 0.01	0.08 ± 0.01	15.7 ± 0.08
‘Kraljevčica’	3.09 ± 0.00	0.17 ± 0.03	18.5 ± 0.02
‘Čelenka’	3.19 ± 0.00	0.13 ± 0.02	14.5 ± 0.07
‘Zelenika’	3.29 ± 0.09	0.10 ± 0.02	15.7 ± 0.06
‘Austrougarka’	3.13 ± 0.09	0.13 ± 0.02	16.1 ± 0.07
‘Bobovec’	3.04 ± 0.08	0.16 ± 0.03	17.8 ± 0.09
‘Kanadska Reneta’	3.29 ± 0.09	0.10 ± 0.02	15.4 ± 0.06
‘Božićnica’	3.25 ± 0.00	0.11 ± 0.02	16.3 ± 0.07
‘Jonagold’	3.24 ± 0.00	0.12 ± 0.02	14.3 ± 0.07
‘Idared’	3.41 ± 0.00	0.07 ± 0.01	15.3 ± 0.07
‘Golden Delicious’	3.41 ± 0.00	0.10 ± 0.02	17.0 ± 0.09
‘Fuji’	3.69 ± 0.02	0.07 ± 0.01	14.1 ± 0.08
‘Granny Smith’	3.14 ± 0.00	0.17 ± 0.03	14.6 ± 0.09

TA—total acids; SDM—soluble dry matter. Mean ± SD based on three juice samples (n = 3).

**Table 2:** Sugar composition of juices produced from old and commercial apple cultivars.

	Sucrose (g/L)	Glucose (g/L)	Fructose (g/L)
‘Wagener’	19.51 ± 0.46 ^g^	15.71 ± 0.29 ^d^	32.36 ± 0.59 ^i^
‘Ilzer Rosenapfel’	23.60 ± 0.26 ^ef^	15.58 ± 0.42 ^d^	27.97 ± 0.34 ^l^
‘Winter Banane’	18.78 ± 0.18 ^g^	10.24 ± 2.03 ^f^	29.52 ± 0.61 ^k^
‘Kraljevčica’	33.56 ± 0.92 ^b^	6.55 ± 0.69 ^g^	35.49 ± 1.14 ^h^
‘Čelenka’	18.42 ± 2.17 ^g^	10.24 ± 0.52 ^f^	31.48 ± 1.05 ^j^
‘Zelenika’	23.49 ± 0.28 ^ef^	17.28 ± 0.06 ^b^	36.23 ± 0.30 ^fgh^
‘Austrougarka’	23.97 ± 0.01 ^ef^	9.92 ± 0.03 ^f^	50.78 ± 0.22 ^a^
‘Bobovec’	32.64 ± 0.13 ^b^	16.14 ± 0.05 ^cd^	49.19 ± 0.10 ^b^
‘Kanadska Reneta’	24.83 ± 1.16 ^de^	13.48 ± 0.06 ^e^	36.08 ± 0.37 ^gh^
‘Božićnica’	22.89 ± 1.49 ^f^	16.97 ± 0.09 ^bc^	41.44 ± 0.25 ^d^
‘Jonagold’	26.10 ± 0.23 ^cd^	4.95 ± 0.30 ^h^	37.04 ± 0.39 ^f^
‘Idared’	26.05 ± 0.21 ^cd^	9.92 ± 0.46 ^f^	39.39 ± 0.48 ^e^
‘Golden Delicious’	39.36 ± 0.65 ^a^	7.56 ± 0.26 ^g^	44.89 ± 0.34 ^c^
‘Fuji’	16.11 ± 1.67 ^h^	19.18 ± 0.00 ^a^	36.84 ± 0.11 ^fg^
‘Granny Smith’	27.29 ± 0.13 ^c^	13.39 ± 0.02 ^e^	36.19 ± 0.09 ^fgh^

Mean ± SD based on three juice samples (n = 3). Within the same column means followed by different letters are significantly different at *p* ≤ 0.05, (ANOVA, Fisher’s LSD).

**Table 3:** Total phenol and flavonoid contents of juices produced from old and commercial apple cultivars.

	TPC (mg/L)	TFC (mg CE/L)
‘Wagener’	164.87 ± 1.70 ^j^	33.23 ± 0.45 ^l^
‘Ilzer Rosenapfel’	159.10 ± 3.57 ^j^	35.68 ± 0.59 ^k^
‘Winter Banane’	257.82 ± 8.33 ^e^	73.72 ± 0.74 ^e^
‘Kraljevčica’	234.74 ± 1.92 ^f^	49.89 ± 1.22 ^h^
‘Čelenka’	115.09 ± 0.98 ^k^	29.21 ± 1.28 ^m^
‘Zelenika’	217.22 ± 5.34 ^g^	57.25 ± 0.45 ^g^
‘Austrougarka’	354.19 ± 4.85 ^c^	87.34 ± 0.34 ^c^
‘Bobovec’	493.72 ± 7.39 ^b^	131.75 ± 0.95 ^b^
‘Kanadska Reneta’	228.33 ± 6.11 ^f^	85.09 ± 1.06 ^d^
‘Božićnica’	501.62 ± 0.74 ^a^	165.58 ± 2.21 ^a^
‘Jonagold’	196.28 ± 3.21 ^h^	44.70 ± 1.45 ^i^
‘Idared’	83.03 ± 2.59 ^m^	18.32 ± 0.59 ^n^
‘Golden Delicious’	99.70 ± 0.98 ^l^	18.52 ± 0.45 ^n^
‘Fuji’	177.48 ± 0.98 ^i^	42.15 ± 0.29 ^j^
‘Granny Smith’	325.34 ± 3.53 ^d^	64.40 ± 0.34 ^f^

TPC—total polyphenol content, TFC—total flavonoid content. Mean ± SD based on three juice samples (n = 3). Within the same column means followed by different letters are significantly different at *p* ≤ 0.05, (ANOVA, Fisher’s LSD).

**Table 4:** The content of flavonols (µg/mL) in juices produced from old and commercial apple cultivars.

	Myricetin	Quercetin-3-Rutinoside	Quercetin-3- D-Glucoside	Quercetin
‘Wagener’	0.53 ± 0.00 ^a^	1.54 ± 0.00 ^b^	0.47 ± 0.01 ^g^	≤LOD
‘Ilzer Rosenapfel’	0.06 ± 0.02 ^i^	0.33 ± 0.01 ^h^	0.35 ± 0.01 ^i^	≤LOD
‘Winter Banane’	0.34 ± 0.00 ^c^	1.07 ± 0.01 ^d^	0.61 ± 0.01 ^f^	0.03 ± 0.03 ^c^
‘Kraljevčica’	0.04 ± 0.00 ^j^	0.56 ± 0.01 ^f^	0.25 ± 0.01 ^j^	≤LOD
‘Čelenka’	≤LOD	0.07 ± 0.00 ^m^	0.04 ± 0.00 ^m^	≤LOD
‘Zelenika’	0.16 ± 0.00 ^e^	0.48 ± 0.01 ^g^	0.75 ± 0.01 ^d^	≤LOD
‘Austrougarka’	0.06 ± 0.00 ^i^	0.54 ± 0.01 ^f^	1.94 ± 0.02 ^a^	0.05 ± 0.02 ^a^
‘Bobovec’	0.10 ± 0.01 ^h^	1.42 ± 0.02 ^c^	1.38 ± 0.01 ^b^	0.03 ± 0.00 ^c^
‘Kanadska Reneta’	0.34 ± 0.01 ^c^	0.22 ± 0.00 ^j^	0.14 ± 0.01 ^k^	0.05 ± 0.00 ^ab^
‘Božićnica’	0.52 ± 0.00 ^b^	1.71 ± 0.01 ^a^	0.72 ± 0.01 ^e^	0.03 ± 0.00 ^c^
‘Jonagold’	0.14 ± 0.00 ^f^	0.23 ± 0.01 ^j^	0.33 ± 0.01 ^i^	0.04 ± 0.00 ^ab^
‘Idared’	0.12 ± 0.00 ^g^	0.10 ± 0.03 ^l^	0.12 ± 0.04 ^kl^	≤LOD
‘Golden Delicious’	0.03 ± 0.00 ^k^	0.16 ± 0.00 ^k^	0.10 ± 0.01 ^l^	≤LOD
‘Fuji’	0.11 ± 0.00 ^g^	0.28 ± 0.00 ^i^	0.40 ± 0.01 ^h^	≤LOD
‘Granny Smith’	0.28 ± 0.01 ^d^	0.61 ± 0.00 ^e^	1.28 ± 0.00 ^c^	0.03 ± 0.00 ^bc^

Mean ± SD based on three juice samples (n = 3). Means with different letters within the same column are significantly different at *p* ≤ 0.05 (ANOVA, Fisher’s LSD). LOD: limit of detection.

**Table 5:** The content of procyanidins (µg/mL) in juices produced from old and commercial apple cultivars.

	Procyanidin A2	Procyanidin B1	Procyanidin B2
‘Wagener’	0.18 ± 0.03 ^f^	94.55 ± 3.05 ^c^	3.28 ± 0.64 ^k^
‘Ilzer Rosenapfel’	0.19 ± 0.02 ^f^	135.32 ± 1.40 ^b^	59.39 ± 3.47 ^g^
‘Winter Banane’	0.69 ± 0.24 ^d^	10.18 ± 2.49 ^f^	26.49 ± 4.14 ^h^
‘Kraljevčica’	0.56 ± 0.11 ^de^	238.05 ± 28.49 ^a^	120.20 ± 6.05 ^e^
‘Čelenka’	0.13 ± 0.00 ^f^	8.55 ± 0.37 ^f^	3.21 ± 0.15 ^k^
‘Zelenika’	0.49 ± 0.04 ^de^	47.58 ± 1.86 ^e^	25.78 ± 0.28 ^h^
‘Austrougarka’	0.16 ± 0.00 ^f^	53.67 ± 7.34 ^de^	236.50 ± 1.16 ^c^
‘Bobovec’	0.21 ± 0.03 ^f^	46.09 ± 10.94 ^e^	422.61 ± 2.01 ^a^
‘Kanadska Reneta’	2.04 ± 0.34 ^a^	13.16 ± 4.01 ^f^	3.20 ± 0.39 ^k^
‘Božićnica’	0.16 ± 0.02 ^f^	50.87 ± 16.21 ^e^	365.31 ± 9.07 ^b^
‘Jonagold’	0.34 ± 0.02 ^ef^	107.12 ± 12.20 ^c^	76.31 ± 0.95 ^f^
‘Idared’	1.68 ± 0.03 ^b^	13.15 ± 0.40 ^f^	10.00 ± 0.08 ^j^
‘Golden Delicious’	1.56 ± 0.11 ^b^	93.49 ± 0.43 ^c^	16.49 ± 0.03 ^i^
‘Fuji’	0.66 ± 0.30 ^d^	67.52 ± 3.03 ^d^	80.19 ± 1.03 ^f^
‘Granny Smith’	1.15 ± 0.04 ^c^	23.18 ± 5.20 ^f^	220.23 ± 6.54 ^d^

Mean ± SD based on three juice samples (n = 3). Means with different letters within the same column are significantly different at *p* ≤ 0.05 (ANOVA, Fisher’s LSD). LOD: limit of detection.

**Table 6:** The content of flavanols and dihydrochalcones (µg/mL) in juices produced from old and commercial apple cultivars.

	Catechin	Epicatechin	Epigallocatechin	Phloridzin
‘Wagener’	2.32 ± 0.01 ^hi^	9.66 ± 0.21 ^de^	6.22 ± 1.32 ^fg^	11.40 ± 0.07 ^b^
‘Ilzer Rosenapfel’	5.71 ± 0.93 ^efg^	6.01 ± 0.22 ^hi^	7.12 ± 0.73 ^f^	7.15 ± 0.05 ^f^
‘Winter Banane’	9.89 ± 1.55 ^c^	10.31 ± 0.48 ^d^	8.21 ± 0.61 ^f^	10.73 ± 0.13 ^c^
‘Kraljevčica’	14.86 ± 1.94 ^b^	13.55 ± 0.53 ^c^	34.38 ± 2.98 ^d^	2.88 ± 0.02 ^i^
‘Čelenka’	3.47 ± 0.05 ^gh^	4.64 ± 0.09 ^i^	≤LOD	1.01 ± 0.02 ^j^
‘Zelenika’	4.20 ± 0.09 ^fgh^	10.82 ± 0.05 ^d^	2.22 ± 0.07 ^gh^	7.06 ± 0.11 ^f^
‘Austrougarka’	6.49 ± 0.77 ^def^	12.34 ± 0.08 ^c^	1.32 ± 0.03 ^h^	5.93 ± 0.03 ^g^
‘Bobovec’	26.86 ± 1.08 ^a^	57.18 ± 1.28 ^a^	46.03 ± 1.67 ^b^	10.15 ± 0.31 ^d^
‘Kanadska reneta’	2.66 ± 0.20 ^hi^	8.59 ± 0.55 ^efg^	6.39 ± 0.26 ^f^	7.16 ± 0.18 ^f^
‘Božićnica’	25.65 ± 4.50 ^a^	24.42 ± 0.70 ^b^	52.87 ± 3.06 ^a^	14.54 ± 0.10 ^a^
‘Jonagold’	8.17 ± 0.36 ^cd^	8.78 ± 0.22 ^ef^	9.05 ± 1.51 ^f^	9.18 ± 0.02 ^e^
‘Idared’	1.04 ± 0.04 ^i^	2.55 ± 0.05 ^j^	≤LOD	0.99 ± 0.01 ^j^
‘Golden Delicious’	0.48 ± 0.11 ^i^	2.61 ± 0.08 ^j^	≤LOD	1.04 ± 0.01 ^j^
‘Fuji’	2.79 ± 0.25 ^hi^	7.30 ± 2.81 ^gh^	14.47 ± 0.62 ^e^	10.62 ± 0.07 ^c^
‘Granny Smith’	7.17 ± 0.69 ^de^	7.43 ± 0.29 ^fgh^	41.06 ± 7.92 ^c^	4.75 ± 0.02 ^h^

Mean ± SD based on three juice samples (n = 3). Means with different letters within the same column are significantly different at *p* ≤ 0.05 (ANOVA, Fisher’s LSD). LOD: limit of detection.

**Table 7:** The content of non-flavonoids (µg/mL) in juices produced from old and commercial apple cultivars.

	Gallic Acid	*trans*-Ferulic Acid	Chlorogenic Acid	Caffeic Acid	*p*-Coumaric Acid
‘Wagener’	4.62 ± 0.75 ^a^	0.14 ± 0.01 ^c^	199.70 ± 0.68 ^f^	0.06 ± 0.00 ^j^	0.04 ± 0.00 ^h^
‘Ilzer Rosenapfel’	4.90 ± 0.16 ^a^	0.03 ± 0.00 ^fg^	123.41 ± 0.64 ^i^	0.57 ± 0.01 ^cd^	0.17 ± 0.00 ^a^
‘Winter Banane’	≤LOD	0.24 ± 0.03 ^b^	353.74 ± 9.93 ^c^	0.60 ± 0.01 ^c^	0.04 ± 0.00 ^gh^
‘Kraljevčica’	2.85 ± 0.42 ^b^	0.04 ± 0.01 ^ef^	244.54 ± 2.22 ^e^	0.32 ± 0.01 ^g^	0.02 ± 0.00 ^k^
‘Čelenka’	≤LOD	0.05 ± 0.00 ^e^	243.23 ± 10.5 ^e^	0.22 ± 0.02 ^hi^	0.12 ± 0.01 ^b^
‘Zelenika’	≤LOD	0.09 ± 0.02 ^d^	182.43 ± 3.11 ^g^	0.46 ± 0.01 ^ef^	0.04 ± 0.00 ^g^
‘Austrougarka’	2.37 ± 0.14 ^c^	0.07 ± 0.00 ^d^	335.02 ± 2.01 ^d^	1.00 ± 0.00 ^a^	0.07 ± 0.00 ^e^
‘Bobovec’	2.37 ± 0.07 ^c^	0.09 ± 0.03 ^d^	563.96 ± 1.56 ^a^	0.40 ± 0.00 ^f^	0.11 ± 0.00 ^c^
‘Kanadska reneta’	≤LOD	0.42 ± 0.00 ^a^	130.95 ± 2.14 ^h^	0.81 ± 0.01 ^b^	0.03 ± 0.00 ^i^
‘Božićnica’	0.36 ± 0.06 ^f^	0.08 ± 0.00 ^d^	418.18 ± 4.67 ^b^	0.53 ± 0.12 ^d^	0.10 ± 0.00 ^d^
‘Jonagold’	1.52 ± 0.47 ^d^	0.02 ± 0.00 ^g^	244.10 ± 0.41 ^e^	0.05 ± 0.00 ^j^	0.02 ± 0.00 ^k^
‘Idared’	0.36 ± 0.02 ^f^	0.09 ± 0.00 ^d^	59.60 ± 0.80 ^l^	0.19 ± 0.00 ^i^	0.03 ± 0.00 ^i^
‘Golden Delicious’	0.85 ± 0.01 ^e^	0.03 ± 0.00 ^fg^	106.12 ± 2.30 ^j^	0.27 ± 0.01 ^gh^	0.06 ± 0.00 ^f^
‘Fuji’	1.52 ± 0.14 ^d^	0.03 ± 0.00 ^efg^	200.29 ± 1.41 ^f^	0.45 ± 0.01 ^ef^	0.03 ± 0.00 ^j^
‘Granny Smith’	0.10 ± 0.08 ^f^	0.02 ± 0.00 ^g^	67.13 ± 0.07 ^k^	0.47 ± 0.01 ^e^	0.06 ± 0.00 ^e^

Mean ± SD based on three juice samples (n = 3). Means with different letters within the same column are significantly different at *p* ≤ 0.05 (ANOVA, Fisher’s LSD). LOD: limit of detection.

**Table 8:** Antioxidant activity measured by two methods DPPH and ABTS expressed as mmol Trolox equivalents/L of juices produced from old and commercial apple cultivars.

	DPPH	ABTS
‘Wagener’	0.18 ± 0.01 ^j^	0.30 ± 0.02 ^g^
‘Ilzer Rosenapfel’	0.16 ± 0.01 ^k^	0.32 ± 0.02 ^g^
‘Winter Banane’	0.33 ± 0.01 ^e^	0.55 ± 0.03 ^d^
‘Kraljevčica’	0.23 ± 0.00 ^h^	0.49 ± 0.01 ^e^
‘Čelenka’	0.14 ± 0.00 ^l^	0.12 ± 0.01 ^h^
‘Zelenika’	0.24 ± 0.00 ^g^	0.57 ± 0.04 ^d^
‘Austrougarka’	0.42 ± 0.01 ^c^	1.00 ± 0.01 ^b^
‘Bobovec’	0.52 ± 0.00 ^a^	1.47 ± 0.03 ^a^
‘Kanadska reneta’	0.20 ± 0.00 ^i^	0.55 ± 0.03 ^d^
‘Božićnica’	0.51 ± 0.00 ^b^	1.45 ± 0.02 ^a^
‘Jonagold’	0.24 ± 0.00 ^gh^	0.45 ± 0.01 ^ef^
‘Idared’	0.08 ± 0.00 ^n^	0.15 ± 0.05 ^h^
‘Golden Delicious’	0.10 ± 0.00 ^m^	0.11 ± 0.02 ^h^
‘Fuji’	0.29 ± 0.00 ^f^	0.42 ± 0.02 ^f^
‘Granny Smith’	0.37 ± 0.00 ^d^	0.86 ± 0.02 ^c^

Mean ± SD based on three juice samples (n = 3). Means with different letters within the same column are significantly different at *p* ≤ 0.05 (ANOVA, Fisher’s LSD).

## Data Availability

The data presented in this study are available on request from the corresponding author. The data are not publicly available due to privacy restrictions.

## Supplementary Materials

The following supporting information can be downloaded at: https://www.mdpi.com/article/10.3390/plants12213733/s1, Figure S1: An example chromatogram for ‘Bobovac’ apple juice.
