# Supplementary figures and images for: Unveiling Apple Diversity: The Quality of Juice Produced from Old vs. Commercial Apple Cultivars
Source: Plants (Basel). 2023 Oct 31;12(21):3733. doi: 10.3390/plants12213733 (PMC10650719; doi:10.3390/plants12213733)

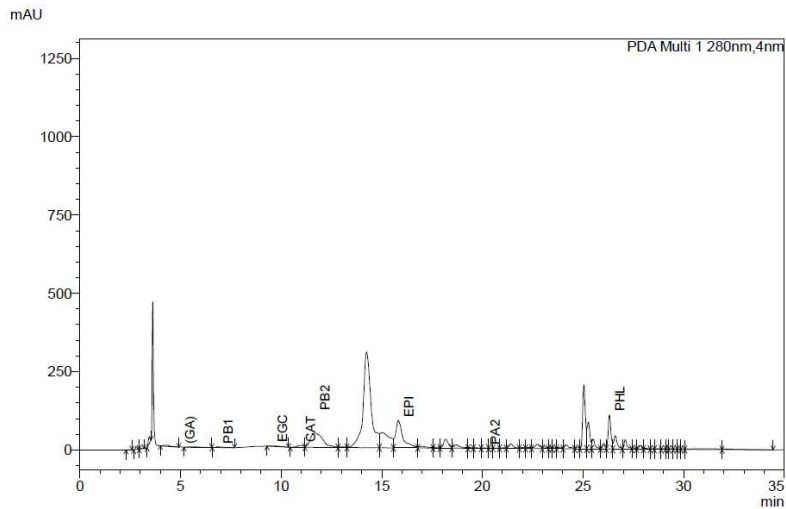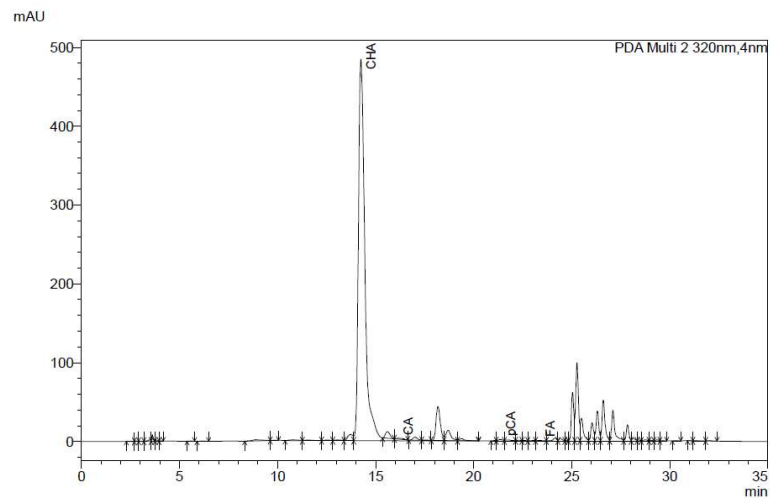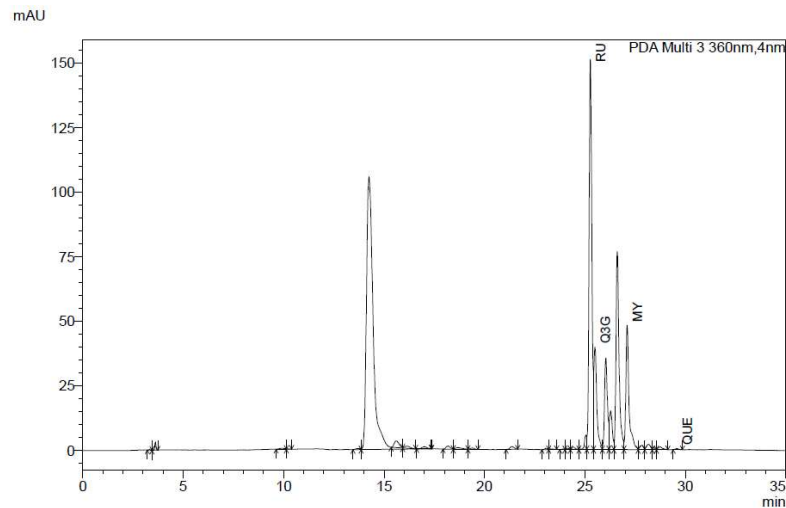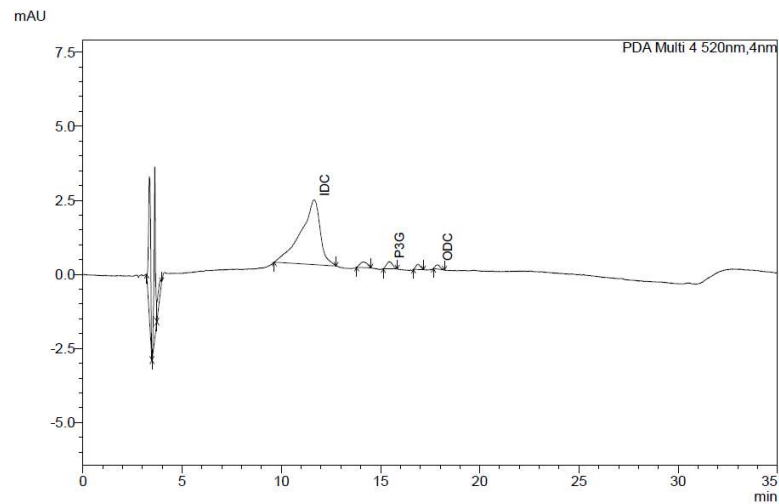

Supplement: Supplementary file 1 [file plants-12-03733-s001.zip › plants-2657218-supplementary.pdf]
